# National Poor-Law Officers' Association, Incorporated

**Published:** 1916-09-09

**Authors:** Tom Percival

**Affiliations:** President, National Poor-Law Officers' Association, Incorporated. Guardians' Hall, North Shields


### National Poor-Law Officers' Association, Incorporated.

*To the Editor of* The Hospital.

Sir,—I very much appreciate the very reasonable statement in your issue of the 19th instant of your views with regard to the efforts of this Association to organise the Poor-Law Nursing Service. Its tone, if you will pardon me saying so, is considerably more attractive than the rather fierce denunciation of this Association and all its works in your previous issue, and encourages me to hope that when you appreciate the reasons which dictate our campaign we shall succeed in enlisting your powerful influence in furtherance of our efforts.

I am not going to waste time and space—yours and mine—by arguing as to the relative correctness of your view of the number of beds in Poor-Law infirmaries, as against my own—the Return is available for anyone interested in the question—and I will merely content myself by pointing out that, apart from the beds in training-schools, the majority of the sick poor occupying the remainder of the beds in Poor-Law infirmaries are being nursed by trained nurses, whose interest in this question is as vital as the staffs of the recognised training-schools.

There is, unfortunately, no gainsaying your statement as to the indifference of a considerable section of our nurses to the claims of the Association to their support, but this indifference is (just as unfortunately) not confined to us, but applies to organisations of every description. That indifference we are steadily breaking down by bringing home to the nurses the importance of the issues at stake and the extent to which we are in a position to be of service to them.

It is equally true that the majority of our probationers have no definite idea of remaining in Poor Law, and that is not a matter for regret, for nobody who realises the need for wide and varied experience desires to see any watertight compartment erected between Poor-Law trained nurses and those trained elsewhere. But it is a fact that the Poor-Law Nursing Service is offering increasing attractions to nurses of high qualifications, and large numbers of these nurses drift back into Poor Law in the course of their after-career, whilst anything affecting the status of the Poor Law generally is bound to affect indirectly the status and value of their Poor-Law certificate.

I do not know on what grounds you state so emphatically that this Association stands for the maintenance of the *status quo*. So far as I am aware no such declaration of policy has ever been made, and whether it would be wise for an organisation of officers to take sides on a question of public policy of this nature is a matter of doubt. Will you consider this point? Whatever you or any of us may consider the wise policy to pursue in regard to the severance of the tie between the Poor Law and the existing form of State hospitals (and there is a good deal to be said on the other side), and whatever likelihood there was of such a separation being brought about before the war, the strain on our financial resources and the attention necessary to the reorganisation of the country at the close of this great struggle, has inevitably put back all such developments for at least a generation. We are dealing with things as they exist to-day. Poor-Law nurses are (willy nilly) members of the great public service to which I have the honour to belong, and whatever is of moment to the service as a whole is of moment to them.

Apart, however, from the general principle, if there were the slightest chance of our nurses effectively organising their strength as a separate body, they would receive every encouragement and assistance from us, but is such a thing practicable? Personally I believe not, and we venture to hold that this Association, numerically strong, possessing considerable influence, with adequate machinery and a Parliamentary Committee which includes many of the leading legal members of our profession, is in a position to render service to our nurses that no separate organisation could possibly hope to secure for many years.

Please accept my apologies for inflicting so long a communication on you, but the advantage which I frankly admit will accrue, can I convince you of the propriety and wisdom of our action, is my excuse.

Filled as your pages are week by week with the story of divine self-sacrifice and unselfish devotion to the needs of others, you should be open to admit the existence of unselfish motives, and I can honestly say that in this matter we have no axe to grind. Our only desire is to be of assistance to a section of our service which commands our admiration and respect, and to secure the interests of all those with whom we have the privilege to serve.—I am, Sir, yours faithfully,

Tom Percival, President,
National Poor-Law Officers' Association,
Incorporated.

Guardians' Hall, North Shields,
August 29, 1916.

